# Supplementary figures and images for: Behavioral signature of trihexyphenidyl in the TOR1A (DYT1) knockin mouse model of dystonia
Source: Dystonia. Author manuscript; Available in PMC 2026 May 29. (PMC13218773; doi:10.3389/dyst.2025.15034)

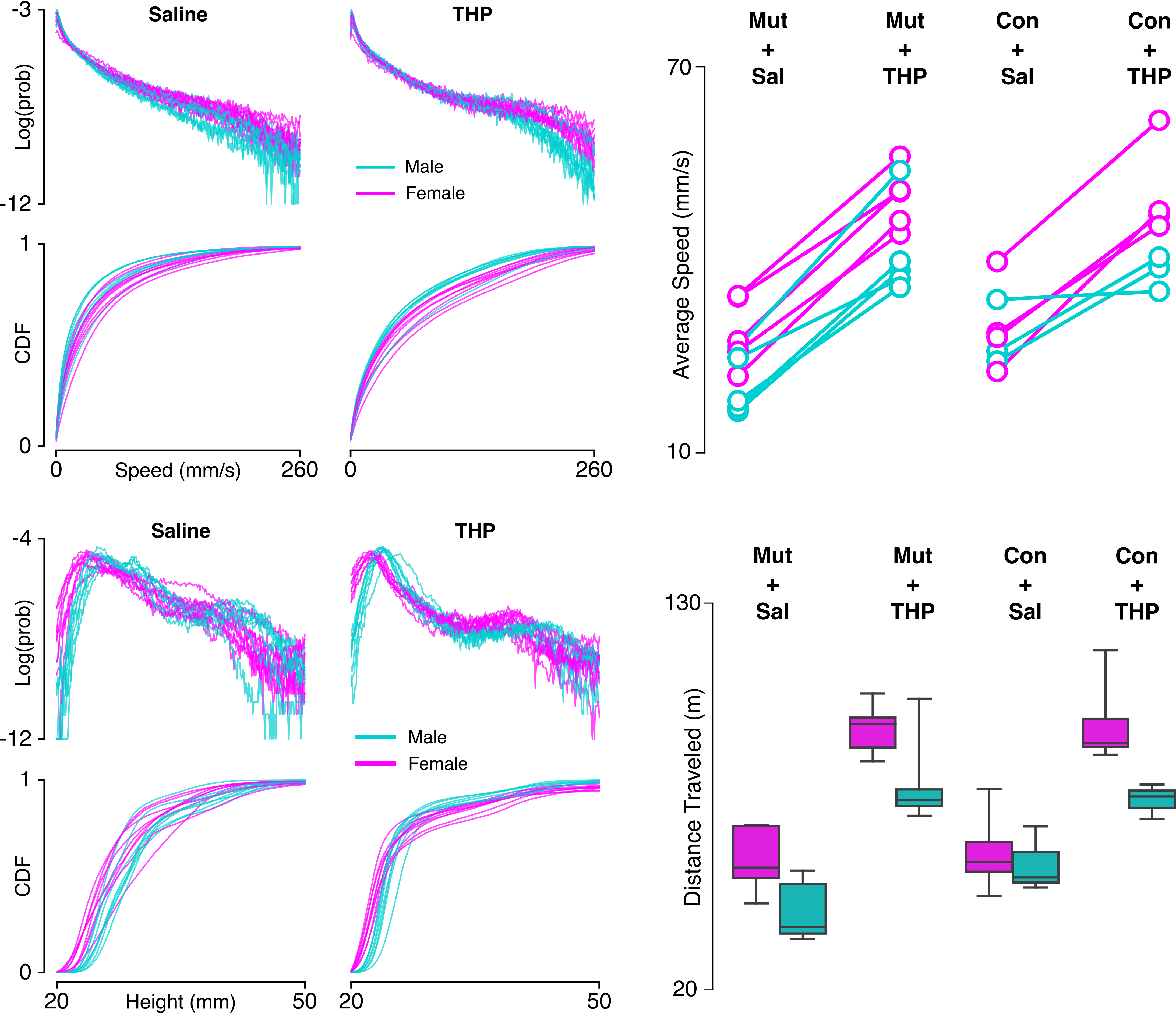

Supplement: supp_fig1 — SUPPLEMENTARY FIGURE S1 Kinematic summaries compared across sex and treatment. (A) Top: The log-transformed probability distribution of the exhibited speed. Bottom: The cumulative distribution function (CDF) of the exhibited speed. The left column shows all saline-treated mice (mutant and control). The right column shows all THP-treated mice (mutant and control). Cyan and magenta represent male and female mice, respectively. The speed distributions suggest that females exhibited generally faster speeds than males after both saline and THP challenge as reflected by the slower initial rise in the speed CDFs of female mice (B) Similar to A, showing the distribution of height values exhibited in each session. (C) Session-averaged speed in control (Con) and mutant (Mut) mice in response to saline or THP challenge. A 3-way ANOVA (genotype × sex × treatment with treatment as the repeated measure) reveals a significant sex × treatment interaction effect (F1,13 = 4.7, p = 0.0492) with post-hoc t tests demonstrating that the difference in average speed was not significant across sex in saline-treated mice (p = 0.0588) but significant in THP-treated mice (p = 0.0031), suggesting that the effect of THP is potentiated in females. (D) Similar to C but with the genotypes collapsed to show sex × treatment interaction. Asterisks indicate statistical significance (p < 0.05). (E) The total distance traveled in control (Con) and mutant (Mut) mice in response to saline or THP challenge. A 3-way repeated measures ANOVA (genotype × sex × treatment with treatment as the repeated measure) demonstrated significant main effects of sex (F1,13 = 8.290, p = 0.0129) whereby females covered a larger distance than males, and treatment (F1,13 = 204.1, p < 0.0001), but no effect of genotype (F1,13 = 0.4094, p = 0.5334). Interaction effects of genotype × sex, genotype × treatment, sex × treatment, or three-way interactions were not observed, (p = 0.8269, p = 0.0549, p = 0.0507, p = 0.0676, respectivel [file NIHMS2178595-supplement-supp_fig1.tiff]

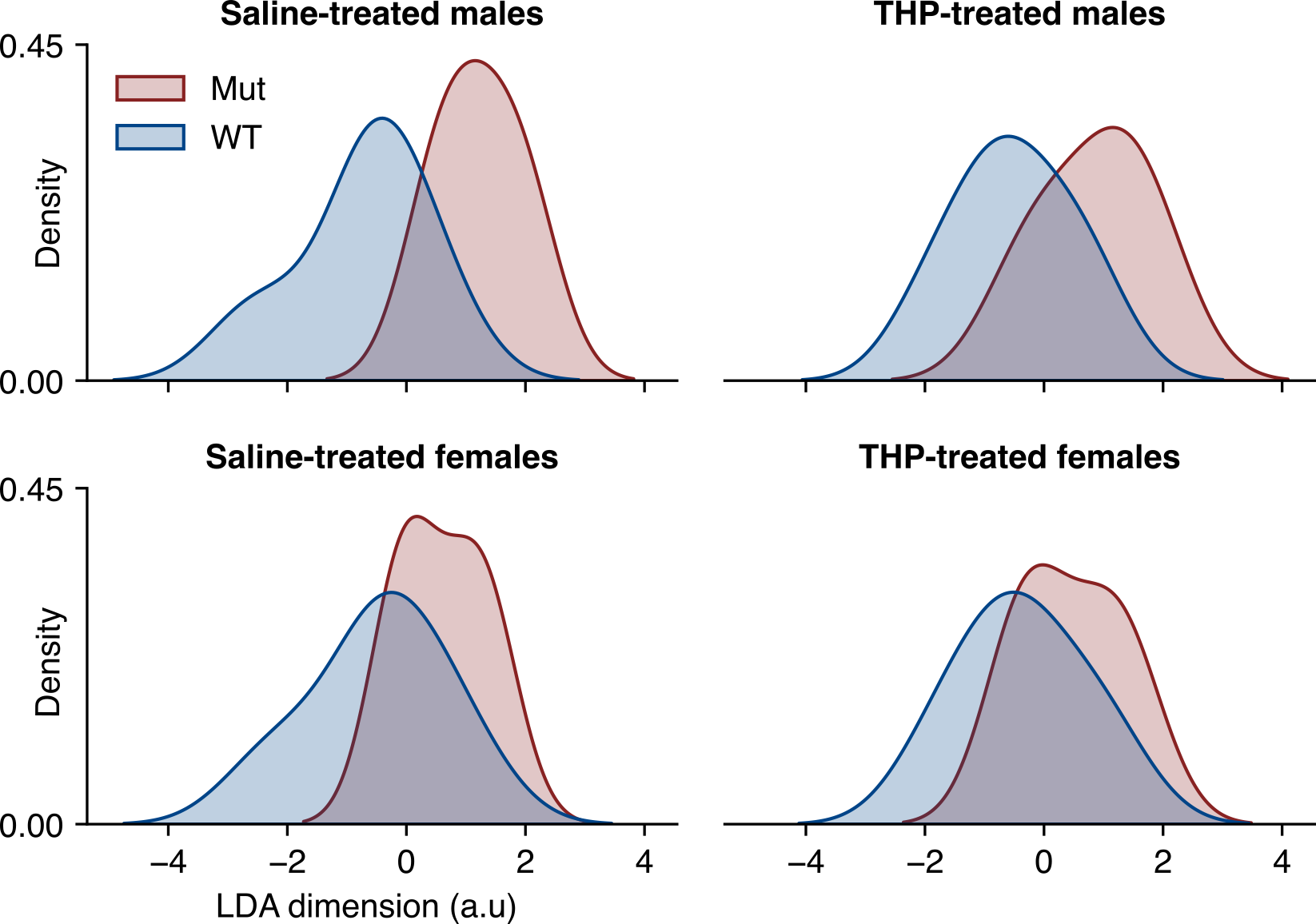

Supplement: supp_fig2 — SUPPLEMENTARY FIGURE S2 Kernel density estimates of syllable usage profiles LDA projection scores. To ensure that THP treatment or sex differences were not diluting a genotype effect in the top PC dimensions, we performed LDA to reduce syllable usage vectors to a single discriminant axis where each sex-treatment combination was projected separately to emphasize any separation of genotypes. Kernel density estimation (KDE) was then applied to smooth the distribution of these projection scores to enable a clear visualization of density patterns and highlighting separation tendencies among the genotypes in each group. [file NIHMS2178595-supplement-supp_fig2.tiff]

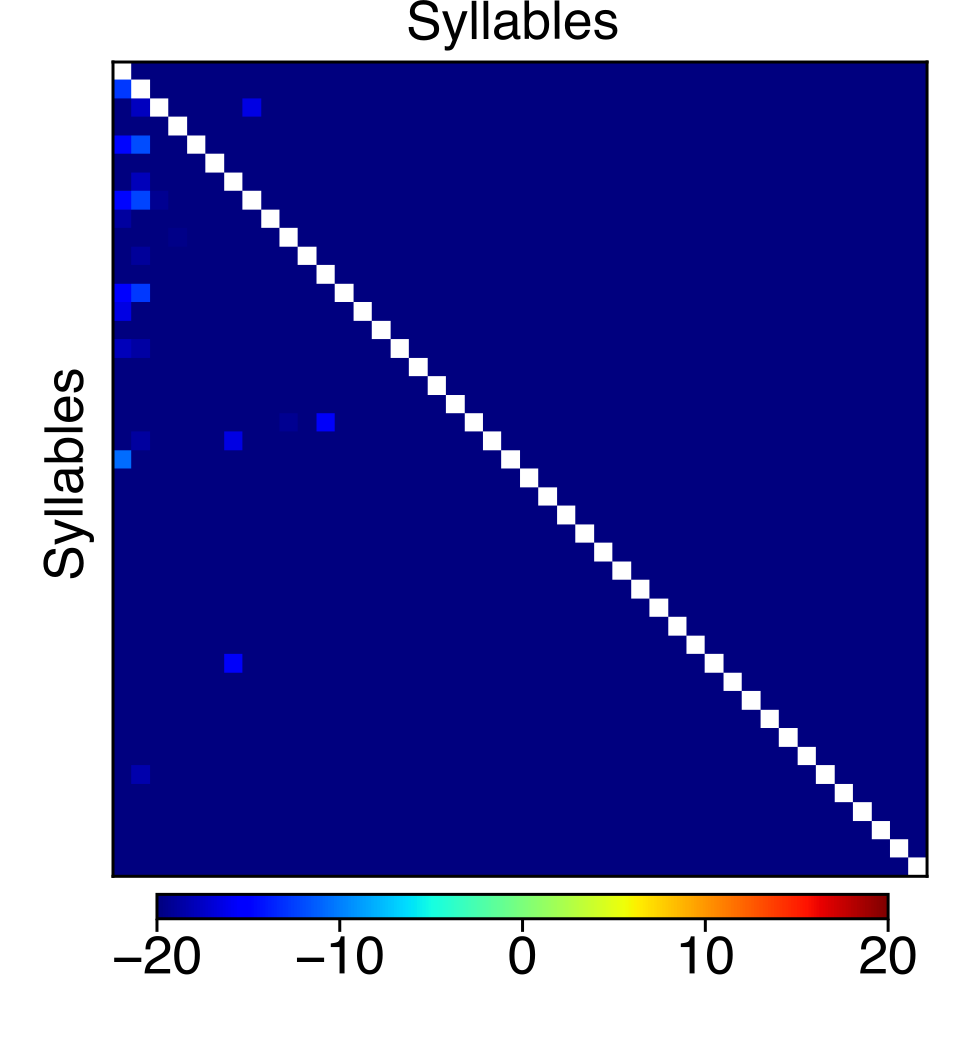

Supplement: supp_fig3 — SUPPLEMENTARY FIGURE S3 Pairwise syllable cross-likelihood analysis. A heatmap showing the cross-likelihood estimate between each pair of identified syllables. The cross-likelihood estimate represents the likelihood that a given occurrence of a syllable is well-modeled by another syllable, thus serving as a validation test of separation of syllables. Values near or above 1 would indicate that one syllable can effectively model another, while values well below 1 would indicate the contrary. Cross-likelihood estimates were calculated as described in Wiltschko et al. [20]. Units are in nats. [file NIHMS2178595-supplement-supp_fig3.tiff]
